# Rapamycin treatment benefits glucose metabolism in mouse models of type 2 diabetes

**DOI:** 10.18632/aging.101117

**Published:** 2016-11-30

**Authors:** Peter C. Reifsnyder, Kevin Flurkey, Austen Te, David E. Harrison

**Affiliations:** ^1^ The Jackson Laboratory, Bar Harbor, ME 04609, USA

**Keywords:** rapamycin, type 2 diabetes, strain survey, insulin sensitivity, pancreatic insulin content

## Abstract

Numerous studies suggest that rapamycin treatment promotes insulin resistance, implying that rapamycin could have negative effects on patients with, or at risk for, type 2 diabetes (T2D). New evidence, however, indicates that rapamycin treatment produces some *benefits* to energy metabolism, even in the context of T2D. Here, we survey 5 mouse models of T2D (KK, KK-Ay, NONcNZO10, BKS-*db/db*, TALLYHO) to quantify effects of rapamycin on well-recognized markers of glucose homeostasis within a wide range of T2D environments. Interestingly, dietary rapamycin treatment did not exacerbate impaired glucose or insulin tolerance, or elevate circulating lipids as T2D progressed. In fact, rapamycin *increased* insulin sensitivity and reduced weight gain in 3 models, and decreased hyperinsulinemia in 2 models. A key covariate of this genetically-based, differential response was pancreatic insulin content (PIC): Models with low PIC exhibited more beneficial effects than models with high PIC. However, a minimal PIC threshold may exist, below which hypoinsulinemic hyperglycemia develops, as it did in TALLYHO. Our results, along with other studies, indicate that beneficial or detrimental metabolic effects of rapamycin treatment, in a diabetic or pre-diabetic context, are driven by the interaction of rapamycin with the individual model's pancreatic physiology.

## INTRODUCTION

Rapamycin increases lifespan in mice and several other organisms [1–8], presumably via inhibition of mTORC (mechanistic Target Of Rapamycin Complex). mTORC activation is associated with the response to nutrients, and it is involved in the regulation of insulin and glucose homeostasis [9–13]. Both glucose and insulin can hyperactivate mTOR, creating a negative feedback loop via S6 kinase to degrade insulin receptor substrate 1/2, impairing insulin signaling, and leading to insulin resistance [14]. Rapamycin can reduce glucose-stimulated insulin secretion and pancreatic islet cell proliferation in mice and in cell lines [reviewed in 9, 10]. Studies of humans taking rapamycin after tissue transplant or as an anti-cancer agent have shown that glucose metabolism is unaffected in most patients; however, a minority (~3–22%) can develop hyper-glycemia, with rates depending on dose of rapamycin, patient population, and individual study [9]. Because rapamycin may alter glucose homeostasis, researchers have evaluated effects of rapamycin on glucose clearance, insulin sensitivity, and adiposity in mice [10–22]. Studies principally used C57BL6 or heterogeneous stocks, but a few have tested models of type 2 diabetes (T2D), such as KK/Hl [15], NONcNZO10 [21], and BKS-*db/db* [17, 18]. In normoglycemic strains, rapamy-cin treatment delays glucose clearance but reduces weight gain and adiposity (particularly when mice are fed a high fat diet). However, rapamycin inconsistently affects insulin sensitivity and serum insulin values. Effects of rapamycin on glucose clearance in already glucose intolerant strains have not been reported, but increased insulin sensitivity has been shown in the insulin resistant BKS-*db/db* mouse [17]. Rapamycin treatment also reduces weight gain in strains that are models of T2D [17, 18, 21]. Furthermore, effects can change over time. In a non-diabetic heterogeneous mouse model, negative effects on glucose clearance and insulin sensitivity faded with treatment duration [13]. It is difficult to compare these studies because of the differences among the methods of rapamycin treatment (intraperitoneal injections or encapsulated in diet), fat content of diets, lengths of treatment, and phenotypic evaluations. Therefore, the present study analyzes the effects of rapamycin across a broad range of diabesity models using a consistent protocol to delineate both common and idiosyncratic responses to the compound. The 5 models selected represent distinct type 2 diabetic etiologies (Table 1), with differing severities of obesity, hyperglycemia, and hyperinsulinemia [23–28].

**Table 1:** Characteristics of the 5 T2D mouse models used in the study

Diabetes strain	Pancreatic insulin content	Hyper-phagia	Obesity	Hyper-insulinemia	Hyper-glycemia	Glucose intolerance	Insulin resistance
KK	High	Yes	Moderate	Severe, by 8 wk	Mild, by 10 wk	Yes	Yes
KK-Ay	High	Yes	Moderate	Very Severe, by 8 wk	Severe, by 16 wk	Yes	Yes
NcZ10	Intermediate	No	Moderate	Mild, by 12–20 wk	Moderate, by 12–20 wk	Yes	Yes
BKS-*db/db*	Low	Yes	Morbid	Severe, by 4–8 wk	Severe, by 4–8 wk	Yes	Yes
TH	Low	No	Moderate	Moderate, by 10–14 wk	Moderate, by 10–14 wk	Yes	Yes

Data references [23–28].

## RESULTS

**Figure 1:**
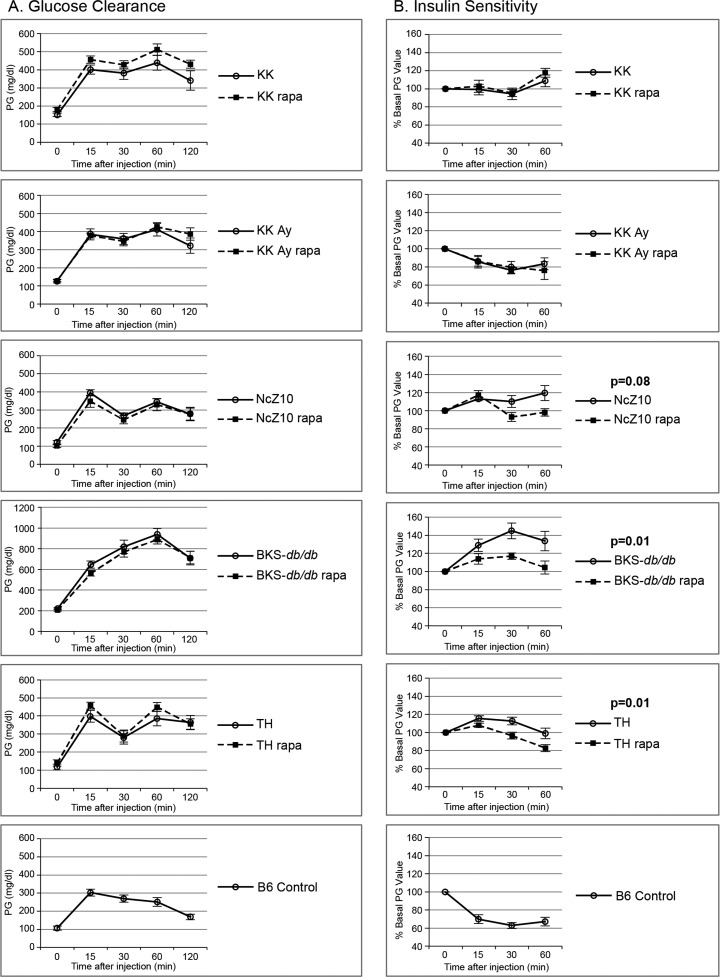
Effect of rapamycin on glucose clearance and insulin sensitivity in 5 diabesity models (**A**) Rapamycin does not exacerbate glucose intolerance in 5 glucose intolerant strains. (**B**) Rapamycin improves insulin sensitivity in the insulin-resistant NcZ10, BKS-*db/db*, and TH strains. Two or three B6 controls were tested with each strain (cumulative data shown, n = 13–16) to serve as positive controls for the glucose and insulin injections, as quality controls, and for reference values. P values are given for repeated measures MANOVA (n = 5–6 per strain/treatment group except for NcZ10, n = 11).

We analyzed effects of rapamycin treatment on 10 T2D phenotypes in 5 models of diabetic mice that represent distinct etiologies. Encapsulated rapamycin was administered through the diet (rapa-treatment), which replicates the alimentary mode of administration used for humans. We evaluated responses, as T2D initially progressed, through 2–6 weeks of rapa-treatment that began at 8–11 weeks of age. Rapa-treatment did not exacerbate the expression of 5 T2D phenotypes (insulin resistance, glucose intolerance, circulating lipids [triglycerides, cholesterol, non-esterified fatty acids]) in any of these diabesity models (Figure 1 and Table 2).

**Table 2:** Rapamycin treatment effects on markers of metabolism in 5 T2D strains

Strain	Group (n)	Glucose clearance (GTT) 3 wk*	Insulin sensitivity (ITT) 2 wk*	Glucose (mg/dl)				Insulin (ng/ml)		TG (mg/dl) fed 6 wk	Cholesterol (mg/dl) fed 6 wk	NEFA (mEq/L) fed 6 wk	Pan-creatic insulin content (ng/mg) 6 wk
				Overnight fasting 3 wk	Fed 2 wk (Glu-2)		Fed 6 wk (Glu-6)	Overnight fasting 3 wk	Fed 6 wk (Ins-6)				
KK	Untreated (6)	Glucose intolerant	Insulin resistant	151 ± 9		190 ± 25	210 ± 14	1.11 ± 0.16	13.9 ± 2.8	388 ± 43	180 ± 2	4.66 ±.24	175 ± 50
	Rapa (6)	No change	No change	176 ± 11		197 ± 23	250 ± 25	3.67 ± 1.56	23.9 ± 9.4	385 ± 96	171 ± 9	4.01 ±.45	167 ± 34
KK-Ay	Untreated (6)	Glucose intolerant	Insulin responsive^**^	126 ± 9		350 ± 47	519 ± 64	1.27 ± 0.12	168.7 ± 34.6	938 ± 129	162 ± 8	5.92 ±.33	161 ± 43
	Rapa (6)	No change	No change	129 ± 5		373 ± 24	418 ± 64	0.83 ± 0.10 **p =.02**	54.6 ± 9.3 **p =.01**	691 ± 140	185 ± 8 **p =.06**	5.44 ±.33	73 ± 8 **p =.06**
NcZ10	Untreated (11)	Glucose intolerant	Insulin resistant	120 ± 11		177 ± 14	218 ± 21	0.45 ± 0.10	1.80 ± .51	286 ± 18	129 ± 5	3.66 ± .11	63 ± 5
	Rapa (11)	No change	Increased sensitivity	102 ± 9		173 ± 10	258 ± 20	0.26 ± 0.03 **p = .10**	1.23 ± .18	249 ± 15	127 ± 5	3.34 ± .10 **p = .06**	43 ± 4 **p = .004**
BKS-*db/db*	Untreated (5–6)	Glucose Intolerant	Insulin resistant	220 ± 11		321 ± 20	638 ± 47	1.81 ± 0.38	7.86 ± 1.15	117 ± 11	207 ± 9	1.92 ± .15	25 ± 4
	Rapa (6)	No change	Increased sensitivity	207 ± 11		375 ± 17 **p = .07**	707 ± 29	1.86 ± 0.23	5.96 ± 1.03	98 ± 7	201 ± 7	1.55 ± .10 **p = .06**	16 ± 2 **p = .08**
TH	Untreated (6)	Glucose intolerant	Insulin resistant	118 ± 15		222 ± 33	365 ± 60	1.79 ± 0.25	4.17 ± 0.84	259 ± 33	226 ± 8	2.97 ± .14	26 ± 6
	Rapa (6)	No change	Increased sensitivity	140 ± 16		278 ± 18	502 ± 16 **p = .05**	1.20 ± 0.25	1.21 ± 0.34 **p = .009**	272 ± 22	247 ± 12	3.31 ± .10 **p = .08**	11 ± 2 **p = .03**

p-values from 1-way ANOVA

* weeks of treatment. See Figure 1 for graphs.

** See Discussion for commentary regarding this result.

**Figure 2:**
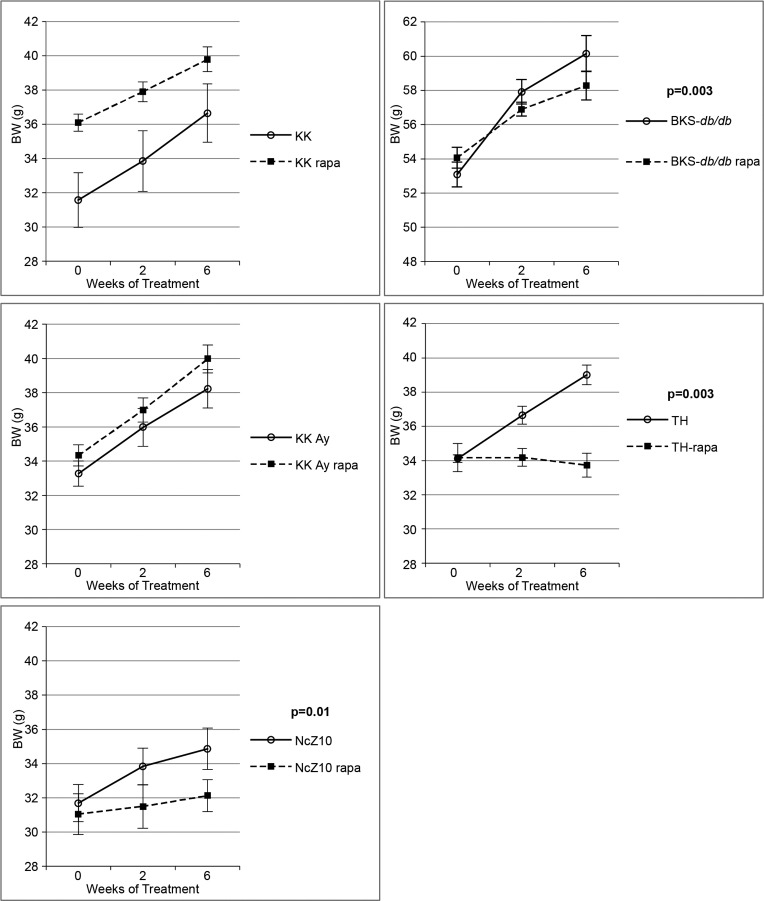
Effect of rapamycin on body weight gain in 5 diabesity models Rapamycin significantly reduces body weight gain in NcZ10, BKS-*db/db*, and TH strains, but not in KK-Ay or KK strains. P values are given for repeated measures MANOVA (n = 5–6 per strain/treatment group, except n = 11 for both NcZ10 groups). Note that the Y-axis scale for BKS-*db/db* is over a different 14-g span than the other 4 strains.

**Figure 3:**
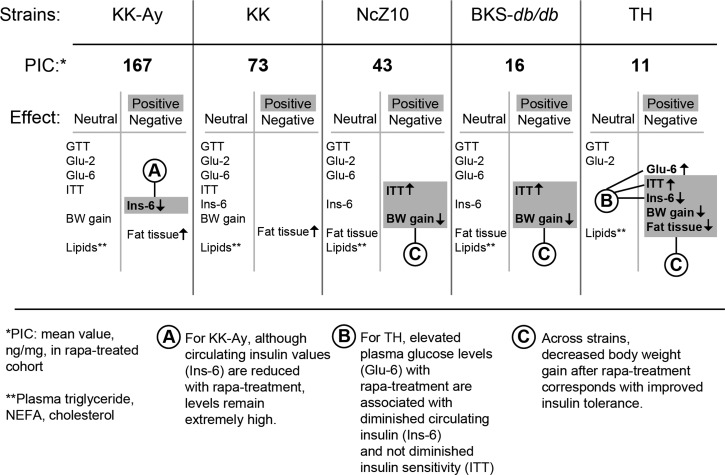
Phenotypic response pattern to rapamycin treatment scales inversely with PIC The balance of positive and negative responses to rapamycin may be shaped by strain differences that control the pancreatic insulin content (PIC) response to rapamycin. The progress of diabetes within the first 6 weeks of treatment is relatively unaffected in strains that retain a relatively high PIC. With intermediate PIC levels, benefits appear that are associated with diminished gain in body weight, including diminished insulin resistance. At the lowest PIC level, glycemic management is impaired and hypoinsulinemic hyperglycemia emerges. *PIC: pancreatic insulin content (ng/mg) after rapa-treatment. **Lipids: plasma triglycerides, NEFA, cholesterol. Terms: GTT = glucose tolerance test; Glu-2 = non-fasting plasma glucose after 2 weeks of treatment; Glu-6 = non-fasting plasma glucose after 6 weeks of treatment; ITT = insulin tolerance test; Ins-6 = non-fasting plasma insulin after 6 weeks of treatment; BW = body weight.

Rapa-treatment elevated hyperglycemia in only one of the 5 models (TH, Table 2). In fact, rapa-treatment produced some benefits. Weight gain was diminished and insulin sensitivity was improved in 3 models (BKS-db/db, NcZ10, TH), and hyperinsulinemia was reduced in 2 models (TH, KK-Ay) (Figure 2, Figure 1B, and Tables 2 & 3). However, rapa-treatment did have a potential negative effect: Pancreatic insulin content (PIC) was diminished in 4 models (significant [p < 0.05] in NcZ10 and TH, suggestive [p = 0.05 to 0.1] in BKS-*db/db*, KK-Ay, Table 2). Results are summarized in Table 4 and Figure 3. Given the expectation that rapamycin would exacerbate disease phenotypes in diabetic models, our results are both surprising and novel.

**Table 3:** Rapamycin treatment effects on adiposity in 5 T2D strains

Strain	Treatment (n)	% Fat pad weight of body weight		
		Epididymal	Retroperitoneal	Inguinal
KK	Untreated (6)	1.75 ± 0.09	0.70 ± 0.05	0.77 ± 0.08
	Rapa (5)	2.18 ± 0.05 **p = .003**	1.25 ± 0.07 **p = .0001**	0.78 ± 0.06
KK-Ay	Untreated (6)	1.36 ± 0.09	0.74 ± 0.07	1.01 ± 0.06
	Rapa (6)	2.47 ± 0.08 **p < .0001**	1.34 ± 0.05 **p < .0001**	0.92 ± 0.09
NcZ10	Untreated (11)	2.25 ± 0.08	0.51 ± 0.04	1.07 ± 0.06
	Rapa (11)	2.03 ± 0.11	0.52 ± 0.04	0.91 ± 0.06 **p = .09**
BKS-*db/db*	Untreated (6)	2.43 ± 0.07	1.42 ± 0.06	3.59 ± 0.14
	Rapa (6)	2.62 ± 0.07 **p = .08**	1.26 ± 0.11	3.62 ± 0.05
TH	Untreated (6)	2.24 ± 0.12	0.49 ± 0.04	1.38 ± 0.08
	Rapa (6)	2.17 ± 0.18	0.35 ± 0.05 **p = .04**	1.05 ± 0.11 **p = .04**

p-values from 1-way ANOVA.

**Table 4:** Summary of effects of rapamycin treatment on T2D-related phenotypes in 5 T2D mouse models

Diabetic strains	Glucose/insulin metabolism					Body composition		Circulating lipids		
	PG elevated	Glucose clearance delayed	Insulin sensitivity increased	Plasma insulin reduced	Pancreatic insulin content reduced	Weight gain reduced	Fat pad weights altered	Serum TG altered	Serum cholesterol altered	Serum NEFA altered
KK	NO	NO	NO	NO	NO	NO	Yes↑	NO	NO	NO
KK-Ay	NO	NO	NO	yes	NO*	NO	Yes↑	NO	NO*	NO
NcZ10	NO	NO	yes	NO	yes	yes	Yes↓	NO	NO	NO*
BKS-*db/db*	NO	NO	yes	NO	NO*	yes	NO	NO	NO	NO*
TH	yes	NO	yes	yes	yes	yes	yes↓	NO	NO	NO*

yes = significant difference (p < .05) between rapa-treated and untreated mice within each strain.

* Suggestive difference, p = .06–.09.

## DISCUSSION

### Specific model-based differences in T2D phenotypes following rapa-treatment

#### KK and KK-Ay

The two KK models that we studied have very high PIC and supraphysiological circulating insulin levels, which distinguishes them from the other 3 models studied.

Rapa-treatment had no effect on insulin resistance in KK and KK-Ay, whereas it diminished insulin resistance in the other 3 models. It is possible that rapa-treatment generally diminishes insulin resistance in diabetic mice, but that sustained, excessive circulating insulin, characteristic of the two KK models, over-whelms this effect. The rapamycin-induced increased adipose tissue weight in the two KK models also contrasted with the response of the other 3 models. The overall 30–40% increase in adipose tissue weight in the KK strains, despite the absence of an increase in food intake or in body weight, suggests that rapa-treatment induced a shift in nutrient partitioning in these models, potentially by *increasing* adipose tissue sensitivity to insulin.

These results stand in contrast to those observed in a previous study of rapa-treatment in high-fat fed KK/HlJ mice [15], a different KK substrain than that used in our study. In both rapa-treated and control KK/HlJ mice [15], mean values of circulating insulin were in the normal range, and, while the authors did not report the glycemic status of their KK/HlJ mice, they did report that their mice maintained a normal glucose clearance after challenge. Results in the KK/HlJ mice studied by Chang et al. [15] may be more typical of our results for NcZ10 and TH than our results for KK and KK-Ay. Like the NcZ10 and TH mice, the KK/HlJ mice studied by Chang et al. had more normal levels of insulin and responded to rapa-treatment with diminished weight gain and lower fat pad weights. Presumably, the high fat-fed KK/HlJ mice model a physiologic state of T2D that provides an environment in which rapa-treatment can have beneficial effects. It may be that the differences between the 2 studies in the responses to rapa-treatment are consequent to an interaction of rapamycin with the dissimilar physiological states established by the large pre-existing differences in insulin levels. Of course, substrain variation or differences in study design and husbandry may be involved.

An unusual characteristic of the KK-Ay strain is that their insulin resistance is not fully reflected by the insulin tolerance test. Insulin resistance is observed in KK mice for adipocyte lipogenesis and suppression of hepatic gluconeogenesis [23, 28]. In contrast, when we evaluated insulin responsiveness using a standard *in vivo* insulin tolerance test, we observed modest insulin responsiveness. We are confident that the circumstances of our test procedure, glucose assay, and insulin preparation were not a cause of this seemingly anomalous insulin responsiveness, as the quality control group (C57BL/6J males) tested at the same time exhibited a normal response to insulin, consistent with the other runs of the insulin tolerance test (ITT) in the study. Furthermore, our observation is consistent with previous reports of modest responsiveness to an insulin tolerance test in KK-Ay mice [29, 30]. The co-existence of even modest insulin sensitivity with the extreme hyperinsulinemia in the same mice is surprising, and it suggests that the yellow obese mutation on the KK genetic background may provide a unique model for elucidation of the mechanisms by which insulin regulates energy metabolism.

#### TALLYHO

PIC in rapa-treated TH mice was significantly reduced to the lowest levels of the 5 models studied. This correlated with a significant decrease in plasma insulin levels and concomitant exacerbation of hyperglycemia, despite the *increased* insulin sensitivity in rapa-treated TH mice. Rapa-treatment also diminished weight gain and adipose tissue weight. This phenotypic pattern in TH — beneficial responses combined with a detrimental response — may be a consequence of the very low PIC levels produced by rapa-treatment specifically in TH mice. Such extremely low PIC levels may cross a threshold for pancreatic maintenance of circulating insulin, resulting in the elevation of circulating glucose. The diminished bodyweight gain and lower fat pad weights in the rapa-treated TH mice may also have resulted from the lower circulating insulin.

#### BKS-db/db

Rapa-treatment tended to diminish PIC in BKS-*db/db* mice to a level similar to, but not quite as low as, that in rapa-treated TH mice. In BKS-*db/db* mice, however, the effect was suggestive but not significant (p = 0.08). While the difference between the models is subtle, it may be critical. In TH mice, plasma glucose levels were significantly increased as plasma insulin decreased, while in BKS-*db/db* mice, both plasma insulin and glucose were unaffected and remained elevated, despite increased insulin sensitivity. This suggests that, as T2D initially progressed in rapa-treated BKS-*db/db* mice, a hypoinsulinemic PIC-threshold had not yet been reached. However, over time, some detrimental effect of rapa-treatment emerges in this model, as lifelong rapa-treatment in BKS-*db/db* mice has been shown to shorten their lifespan [31]. In untreated BKS-*db/db* mice circulating insulin progressively declines over time [32], and we have confirmed that plasma insulin values decrease at the same rate in both untreated and rapa-treated BKS-*db/db* mice with age (unpublished). Given the tendency toward diminished PIC in rapa-treated BKS-*db/db* mice after 6 weeks, we speculate that rapa-induced PIC depletion accelerates the inherent model-driven islet malfunction to further advance the progression to hypoinsulinemia.

Weight gain in the BKS-*db/db* mice was reduced with rapa-treatment, an effect also seen in previous studies [17, 18, 29]. The effect is subtle at 6 weeks of treat-ment, at which time fat pad weights were unaffected in our study. A longer treatment-duration, such as the 6-month treatment used by Deepa et al. [17], may be needed to observe effects directly on adipose tissue.

#### NcZ10

PIC in NcZ10 mice is intermediate between that of the two KK models and the TH and BKS-*db/db* models. Rapa-treatment significantly reduced PIC in NcZ10 mice. Insulin resistance, weight gain, and adipose tissue weight were diminished, as in the TH model; but, in contrast, circulating insulin levels were not diminished and plasma glucose was not elevated. Apparently, the diminished PIC value after rapa-treatment in this model remains sufficient to sustain the pre-treatment relationship of circulating insulin to plasma glucose.

A comparison of our present, 6-week study to a previous, 14-week, rapa-treatment study of NcZ10 males from our laboratory [21] suggests the importance of treatment duration on some outcomes. Diminished weight gain was observable by 6 weeks of treatment in both studies, and suppression of weight gain continued with further rapa-treatment [21]. Although, in both studies, plasma glucose was unaffected at 6 weeks of rapa-treatment, continued treatment elevated plasma glucose [21]. This further elevation of hyperglycemia in the rapa-treated mice was associated with diminished circulating insulin, suggesting that a hypoinsulinemic-associated hyperglycemia can develop over time with rapa-treatment. Similarly, plasma cholesterol (but not plasma triglycerides or non-esterified fatty acids) also was elevated by 14 weeks of treatment in the previous study, but not at 6 weeks of treatment in the present study. These observations suggest that specific effects of rapa-treatment on glucose and lipid metabolism may appear only with long-term treatment.

## CONCLUSION

Rapamycin has extraordinary potential as a treatment for a wide range of chronic diseases. It is already being prescribed to suppress tissue rejection or graft vs. host reactions in patients with tissue and bone marrow transplants. It also is being investigated for treatment of specific cancers and autoimmune diseases [9]. Furthermore, rapamycin is the first drug found to reliably increase maximum lifespan in a mammalian model [1, 2], suggesting that numerous age-related diseases may respond to rapamycin treatment [33]. But an important concern regarding rapamycin treatment is the elevation of circulating glucose observed in some patients. Initial conjecture that this elevation is due to rapamycin-driven insulin resistance [reviewed in 10, 11, 14] is presently tempered by a growing body of evidence that, depending upon the mouse model, rapamycin can have no effect on insulin resistance [12, 13] or can actually promote insulin sensitivity [17, 34]. The foremost finding of our study is that 6 weeks of rapa-treatment does not promote further insulin resistance, glucose intolerance or hyperlipidemia in the diabetic physiologic context represented by multiple T2D mouse models. On the contrary, a number of beneficial effects appeared among some of the T2D strains; the most common were diminished gain in body weight and improved insulin sensitivity.

The potential beneficial effects of rapa-treatment contrast with a potential detrimental effect on PIC. Genetic differences in the regulation of PIC and its response to rapamycin may be key in determining whether a beneficial phenotypic response profile is produced (Figure 3). In diabetic strains with high PIC, rapa-treatment may produce minimal beneficial effects, primarily due to sustained supraphysiologic levels of circulating insulin. In diabetic strains with intermediate or low PIC, rapa-treatment may diminish weight gain and adiposity. The reduced expression of such pathogenic physiological phenotypes with rapamycin treatment could contribute to the associated protection from numerous pathologic sequalae of T2D, including retinopathy, [34], nephropathy [21] and cardiomyopathy [18]. Such observations are consistent with the idea that elevations of circulating insulin levels in diabetic states may play a greater role in the development of T2D-associated lesions than currently appreciated [35].

In mice at risk for or expressing T2D, however, the pre-existing diabetogenic Δ-cell stress interacts with an additional stress associated with initial rapamycin treatment (reviewed in 36) to potentially exacerbate T2D-driven Δ-cell impairment. Thus, the potential for rapamycin-facilitated pathogenesis may be determined by the balance between the pre-existing T2D suscep-tibility and the rate of adjustment to rapamycin-driven Δ-cell stress. If Δ-cell impairment reaches a critical threshold, indicated by extreme PIC depletion, a terminal hypoinsulimic crisis can result. This model is consonant with an emerging emphasis on the role of pancreatic Δ-cell physiology in shaping the risk for, and progression of, T2D [37].

We propose that the effect of long-term rapa-treatment on survival in individuals at risk for, or expressing, T2D is determined by the balance of beneficial effects on insulin responsiveness, body composition, and T2D-associated diseases against the detrimental effect on PIC itself. Future research should emphasize investigation of means to ameliorate detrimental effects of rapamycin on PIC while maintaining the positive effects on other tissues. Resolution of this challenge could provide a major advance in the treatment of complex degenerative diseases.

## METHODS

### Animals

The study comprised males of 5 classic “diabesity” models of mice, all obtained from the Jackson Laboratory at 7–10 weeks of age. The models were: BKS.Cg-*Dock7^m^* +/+ *Lepr^db^*/J (BKS-*db/db*), KK.Cg-*a/a*/J (KK; normal, wild-type nonagouti), KK.Cg-*A^y^*/J (KK-Ay; agouti yellow), NONcNZO10/LtJ (NcZ10), and TALLYHO/JngJ (TH). At 8–11 weeks of age, the mice were separated into 2 groups of 6, weighed, and put onto diet (5LA0, 11% fat, Purina) with or without encapsulated rapamycin (14 PPM, [1]). All mice were fed *ad lib* and given acidified water. Mice were housed in double pen boxes (3 mice per side) with pine shaving bedding. The mouse room was maintained at ~25°C and 40–50% humidity. The specific pathogen free health status of the room can be viewed at (http://myjax.jax.org/lahs/healthreports/RAF/d1.0814.pdf). Two separate cohorts of the NcZ10 strain (5 or 6 mice per treatment group) were studied 3 months apart. The second cohort weighed 3.5 g more than the first cohort at the beginning of treatment, but body weights between the two cohorts, within each treatment group, did not differ at the end of the experiment. The 2 cohorts did not differ for any phenotypic response to rapamycin. All other groups were studied in single cohorts.

### Protocols

After 2 weeks on diets ± rapamycin, an insulin tolerance test (ITT) was performed. Food was removed from the mice at 7:00 a.m. At ~10:00–10:30, mice were weighed and bled from the tail (~2 μl from a nick in the tail tip), glucose was measured (OneTouch Ultra, Lifescan), and mice were then injected i.p. with 1.0 U/kg insulin (Humulin, Eli Lilly) in PBS. Glucose was measured additionally at 15, 30, and 60 minutes post injection. After 3 weeks of treatment, a glucose tolerance test (GTT) was performed. At ~5:00 p.m. on the day before the test, the mice were moved to clean cages and food was removed for overnight fasting. At ~10:00–10:30 a.m. the following morning, the mice were weighed, bled for glucose measurement, and injected with 1 g/kg glucose from a 10% glucose solution in PBS. Glucose was then measured at 15, 30, 60, and 120 minutes. For the 0- and 15-minute time points, the mice were bled by retro-orbital sinus to collect enough blood to measure plasma insulin by ELISA (Meso Scale Discovery). For the remaining time points, blood was collected from the tail tip. For each day that we performed a GTT or an ITT, 2–3 age-matched B6/J males on 4% fat irradiated diet (5LG6, Purina) were included in the testing as positive controls, quality controls, and for reference values. The mean values for each GTT and ITT of the quality control groups, expressed as the percent change from the initial value, were comparable across all runs (n = 5 for the GTT; n = 6 for the ITT; p = 0.12 for the difference among runs for both the GTT and ITT [repeated measures MANOVA]). After 4–5 weeks on diets, food consumption over periods of 3–7 days was measured: the grain in the hopper was weighed before and after the allotted time; after the allotted time, the bedding was sifted and the weight of the wasted food was subtracted from the weight of the food removed from the hopper. Rapamycin did not decrease food intake for any strain. Mice were sacrificed after 6 weeks on the diets. The mice were weighed and bled for sera by retro-orbital sinus immediately before sacrifice by cervical dislocation. Pancreas and a portion of the liver from each mouse were frozen in liquid nitrogen and stored at −80°C. Epididymal, retroperitoneal, and inguinal fat pads were weighed and frozen in liquid nitrogen and stored at −80°C. Pancreatic Insulin Content (PIC) was determined by extracting the insulin from the weighed pancreas by homogenization in acid/ethanol (1.5% HCl, 70% EtOH); insulin con-centration was determined by ELISA (Meso Scale Discovery, Gaithersburg, MD, USA). Aliquots of acid/ethanol samples were neutralized in an equal volume of 1M Tris (pH 7.5) before further dilution (50 fold) in “Diluent 100” provided in the ELISA kit. Sera from sacrifice were measured for glucose, triglycerides, total cholesterol, and non-esterified free fatty acids using the UniCel DxC 600 Synchron clinical system (Beckman Coulter, Inc., Brea, CA, USA), and for insulin by ELISA (MSD).

### Statistical analysis

ANOVA (JMP, SAS Institute, Inc., Cary, NC, USA) was used for within-strain comparisons for effect of treatment. Repeated measures MANOVA (JMP) was used to determine within-strain effects of treatment on ITT, GTT and body weight gain.
